# Prevalence and characteristics of cutaneous allodynia in probable migraine

**DOI:** 10.1038/s41598-021-82080-z

**Published:** 2021-01-28

**Authors:** Seung Min Han, Kyung Min Kim, Soo-Jin Cho, Kwang Ik Yang, Daeyoung Kim, Chang-Ho Yun, Min Kyung Chu

**Affiliations:** 1 Department of Neurology, Severance Hospital, Yonsei University College of Medicine, 50-1 Yonsei-ro, Seodaemun-gu, Seoul, 03772 Republic of Korea; 2 Department of Neurology, Yongin Severance Hospital, Yonsei University College of Medicine, Yongin, Korea; 3 Department of Neurology, Dongtan Sacred Heart Hospital, Hallym University College of Medicine, Hwaseong, Korea; 4 Department of Neurology, Soonchunhyang University College of Medicine, Cheonan Hospital, Cheonan, Korea; 5 Department of Neurology, Chungnam National University Hospital, Daejeon, Korea; 6 Department of Neurology, Bundang Clinical Neuroscience Institute, Seoul National University Bundang Hospital, Seongnam, Korea

**Keywords:** Neurology, Risk factors

## Abstract

Cutaneous allodynia (CA) is a pain in response to non-nociceptive stimulation and a marker of central sensitisation. Probable migraine (PM) is a migraine subtype that fulfils all but one criterion of migraine. Headache intensity and the disability of individuals with PM are similar or lower than individuals with migraine. This study compared CA prevalence and characteristics of PM and migraine using a nationally representative sample in Korea. The Allodynia Symptom Checklist-12 (ASC-12) was used to assess CA (ASC-12 score ≥ 3). PM and migraine prevalence were 11.6% and 5.0%, respectively. CA prevalence did not significantly differ between PM and migraine (14.5% vs. 16.0%, *p* = 0.701). Participants with PM with CA reported a higher monthly headache frequency (3.3 ± 4.3 vs. 1.8 ± 3.6, *p* = 0.044), more severe headache intensity (Visuals Analogue Scale, 6.0 [4.0–7.0] vs. 5.0 [3.0–6.0], *p* = 0.002), and higher impact of headache (Headache Impact Test-6, 56.3 ± 7.2 vs. 48.3 ± 8.0, *p* < 0.001) than those without CA. Multiple regression analyses revealed that headache frequency and intensity, anxiety, and depression were significant factors for CA in participants with PM. In conclusion, CA prevalence among participants with PM and migraine were comparable. Anxiety, depression, and headache frequency and intensity were significant factors for CA in participants with PM.

## Introduction

Cutaneous allodynia (CA) refers to a pain provoked by stimulation of skin that would ordinarily not produce pain^1^. The underlying mechanism of CA includes sensitisation of the trigeminal nucleus caudalis, which receives afferent input from the meninges and periorbital skin regions^2,3^. Central sensitisation is a manifestation of increased excitability of neurons in the central nociceptive pathways, and CA is a clinical marker of central sensitisation^2–4^. It has been reported that a significant proportion of individuals with migraine experience CA during episodes of headache^5–7^. Individuals with migraine combined with CA were reported to have poorer response to acute treatment and a higher rate of progression to chronic migraine (CM) and more severe disability compared to individuals with migraine alone^5,8^. CA in individuals with migraine was associated with comorbid pain conditions, such as irritable bowel syndrome, fibromyalgia (FM), and chronic fatigue syndrome^9^. High attack frequency, depression, and obesity were reported to be significant factors of CA in these individuals; therefore, CA provides clues on the pathophysiology of migraine^7^.

The gold standard for the assessment of CA at any particular point in time is quantitative sensory testing (QST)^10^. Pain thresholds for heat, cold, and pressure stimuli can be assessed by QST^11^. Nevertheless, CA evaluation using QST is difficult in a clinical setting. The Allodynia 12-item questionnaire (Allodynia Symptom Checklist-12, ASC-12) was developed and validated in a large migraine population^5^. It showed a close correlation with frequency and headache intensity as well as migraine-related disability^12^.

Probable migraine (PM) is a subtype of migraine that fulfils all but one criterion of migraine^13^. Previous studies found that 5–15% of the general population experienced PM during the preceding year, and headache intensity and the related disability were comparable or lower among individuals with PM than among those with migraine^14–17^. A study conducted in the USA reported that the prevalence of CA in PM was slightly lower than that in migraine^7^. However, there is currently limited information on the associated factors and characteristics of CA in individuals with PM in a population-based setting. We hypothesised that, as with migraine, headache frequency, depression, and obesity were significant factors for CA in PM. The present study aimed to investigate the prevalence, characteristics, and associated factors of CA in individuals with PM and in those with migraine using the ASC-12.

## Methods

### Survey

We used data derived from the Korean Sleep-Headache Study (KSHS), a nation-wide population-based cross-sectional survey on headache and sleep. A two-stage stratified clustered sampling method was used in the KSHS. Korea is composed of 16 regions (one special city, six metropolitan cities, and nine provinces), which were designated as primary sampling units, except Jeju-do, in the first stage. In the second stage, we selected representative basic administrative units (cities, counties and districts) from each primary sampling unit. Overall, 60 representative basic administrative units were selected from 226 basic administrative units for this study. We assigned a target sample size based on age, sex, and occupation to each selected basic administrative unit as per data from the 2016 population and housing census by the National Statistical Office^18^. We targeted 2500 individuals aged ≥ 19 years. The estimated sampling error was ± 1.9%. The survey was conducted via door-to-door visits and face-to-face interviews using questionnaires by trained interviewers. All interviewers were employees of Gallup Korea and were not medical personnel. The KSHS survey was conducted between October 2018 and November 2018.

### PM and migraine diagnosis

PM was diagnosed according to the third edition of the International Classification of Headache Disorders (ICHD-3; code 1.5)^13^. If the characteristics and accompanying symptoms of a participant’s headache fulfilled all but one of the A–D criteria of migraine without aura, a diagnosis of PM was established.

Migraine was diagnosed based on the diagnostic criteria for migraine without aura according to code 1.1 of ICHD-3^13^. If the characteristics and accompanying symptoms of a participant’s headache fulfilled the A–D criteria of migraine without aura, a diagnosis of migraine was established. We previously evaluated the diagnostic ability of our questionnaire for migraine by comparing the diagnosis using the questionnaire and diagnosis made by neurologists with an additional telephone interview^16^. We did not separately analyse participants according to the presence of aura; therefore, migraine included both migraine with aura (ICHD-3 code 1.2) and migraine without aura (ICHD-3 code 1.1) in the present study. Accordingly, PM included both PM with aura (ICHD-3 code 1.5.2) and PM without aura (ICHD-3 code 1.5.1).

### Assessment of CA

We investigated CA using the 12-item Allodynia Symptom Checklist (ASC-12). The ASC-12 measures interictal CA over the previous month using 12 questions on the thermal, mechanical static, and mechanical dynamic symptoms of CA^5^. Participants were asked to rate 12 questions using any of the following responses: ‘does not apply to me’; ‘never’; ‘rarely’; ‘less than half the time’; and ‘half the time or more’. The first three responses were scored as 0, while ‘less than half of the time’ was scored as 1, and ‘half the time or more’ was scored as 2. The sum of the scores in the 12 items of ASC-12 was the ASC-12 score. If the ASC-12 score was ≥ 3, the participant was identified as having CA. The ASC-12 scores were further sub classified into mild (score 3–5), moderate (score 6–8), and severe (score ≥ 9) CA, respectively^6^.

### Impact and disability of headache

The impact of headache was assessed using the Headache Impact Test-6 (HIT-6)^20^. We used the Korean version of HIT-6, which was previously validated in the Korean language^21^. We used the migraine disability assessment (MIDAS) questionnaire to assess headache-related disability^22^. The MIDAS questionnaire is composed of five questions on the loss of or decrease in productive days due to headache during the previous 3 months. The MIDAS questionnaire was previously validated in the Korean language with good sensitivity and specificity^23^.

### Assessment of depression and anxiety

Depression was assessed by the Patient Health Questionnaire-9 (PHQ-9)^24^. Participants with PHQ-9 scores of 10 or more were defined as having depression. The Korean version of the PHQ-9 has been evaluated showing a sensitivity of 81.1% and a specificity of 89.9%^25^.

We used the Generalized Anxiety Disorder-7 (GAD-7) questionnaire to evaluate anxiety^26^. A participant giving a positive response to seven or more items was classified as having anxiety. The Korean version of GAD-7 was validated with a sensitivity of 78.1% and a specificity of 74.6%^27^.

### Ethical approval

This study was approved by the Institutional Review Board of Severance Hospital, Yonsei University (approval No. 2019–1721-001). Written informed consent was obtained from all participants Prior to obtaining written informed consent, all participants were given an explanation on the objective of the study and the data to be collected by interviewers. All procedures involving human participants were in accordance with the ethical standards of the institutional and/or national research committee as well as the tenets of the 1964 Declaration of Helsinki and its later amendments, or comparable ethical standards.

### Statistical analyses

The 1-year prevalence of migraine was calculated as the number of cases per 100 persons. Binary and ordinal scales are represented as numbers and percentages. Interval scales are represented as mean and standard deviation or median and interquartile range (IQR) when appropriate. Normality of the ratio of variables was tested with the Kolmogorov–Smirnov test. After normality was confirmed, independent two-tailed t-tests or one-way analyses of variance were used to compare the variables between the groups when appropriate. For non-normally distributed variables, a two-tailed Mann–Whitney *U* test or Kruskal–Wallis test was used. To compare interval variables between the groups, we used a Mann–Whitney *U* test or Kruskal–Wallis test and summarised the data as median (IQR). A two-tailed chi-squared test was used to compare the binary and ordinal scales. No statistical power calculation was conducted before commencing the study, and the sample size used was based on the available data. Post hoc comparisons were performed using a Bonferroni's correction (*p* = 0.05/3, 0.017) to adjust for multiple testing among the three groups.

Since the prevalence of PM and migraine varied significantly according to sex and age, we compared clinical characteristics of PM and migraine according to the presence of CA with age and sex adjustment^16,28^. Logistic regression analyses after age (year, continuous) and sex adjustment were used to compare categorical variables according to the presence of CA in PM and migraine. Linear regression analyses with age and sex adjustment were used to compare numerical variables.

We evaluated the odds ratios with 95% confidence interval to determine the factors contributing to CA using univariable and multivariable logistic regression analyses. In individuals with migraine and PM, factors that demonstrated significant differences between those with CA and those without CA were included in the univariable analyses. For multivariable analyses, sociodemographic variables (age, sex, and educational level), headache-related parameters (the intensity and frequency of headache) and psychiatric conditions (anxiety [GAD-7] and depression [PHQ-9]) were included to examine the association of CA with migraine and PM.

SPSS v24.0 (IBM, Armonk, NY, USA) was used to perform the statistical analyses. Statistical significance was set at two-tailed *p* < 0.050. The current analysis is the primary analysis for prevalence of CA in PM using KSHS data. Imputation techniques were not used to minimise non-response effects. The approach used in this study will produce slightly larger standard errors in finite samples when compared with imputation techniques^29^.

## Results

### Survey

Overall, 2501 participants completed the survey. The distribution of sex, age, and educational level among our participants did not significantly differ from that of the total population of Korea (Table 1). Therefore, there was no need to use extracted sampling weights in the statistical analyses.

**Table 1:** Sociodemographic distribution of survey participants and participants identified as having probable migraine and migraine in the 2018 survey as proportions of the overall population.

	Survey participants, N (%)	Total population N (%)	*p*-value	Probable migraine, % (95% CI)	Migraine, % (95% CI)
**Sex**					
Male	1242 (49.6)	21,024,909 (48.9)	0.977	9.8 (8.2–11.5)	4.0 (2.9–5.1)
Female	1259 (50.4)	21,544,156 (50.1)		13.3 (11.4–15.1)	6.0 (4.7–7.3)
**Age, years**					
19–29	434 (17.3)	7,624,184 (17.8)	1.000	11.1 (8.1–14.0)	4.3 (2.4–6.3)
30–39	425 (17.0)	7,446,677 (17.3)		8.9 (6.2–11.7)	7.8 (5.2–10.3)
40–49	498 (19.9)	8,408,883 (19.6)		13.7 (10.6–16.7)	4.8 (2.9–6.7)
50–59	498 (19.9)	8,515,725 (19.8)		12.0 (9.2–14.9)	4.6 (2.8–6.5)
60–69	345 (13.8)	5,854,493 (13.6)		11.6 (8.2–15.0)	4.4 (2.2–6.5)
> 70	301 (12.0)	5,082,994 (11.8)		11.6 (7.9–15.3)	3.7 (1.6–5.8)
**Educational level**					
Middle school or less	393 (15.7)	5,194,888 (12.1)	0.607	10.9 (7.8–14.0)	4.1 (2.1–6.0)
High school	1063 (42.5)	17,130,249 (39.9)		12.2 (10.3–14.2)	4.9 (3.6–6.2)
College of more	1045 (41.8)	20,607,819 (48.0)		11.1 (9.2–13.0)	5.4 (4.1–6.8)
**Total**	2501 (100.0)	42,932,956 (100.0)		11.6 (10.3–12.8)	5.0 (4.1–5.9)

CI: confidence interval.

**Figure 1.**
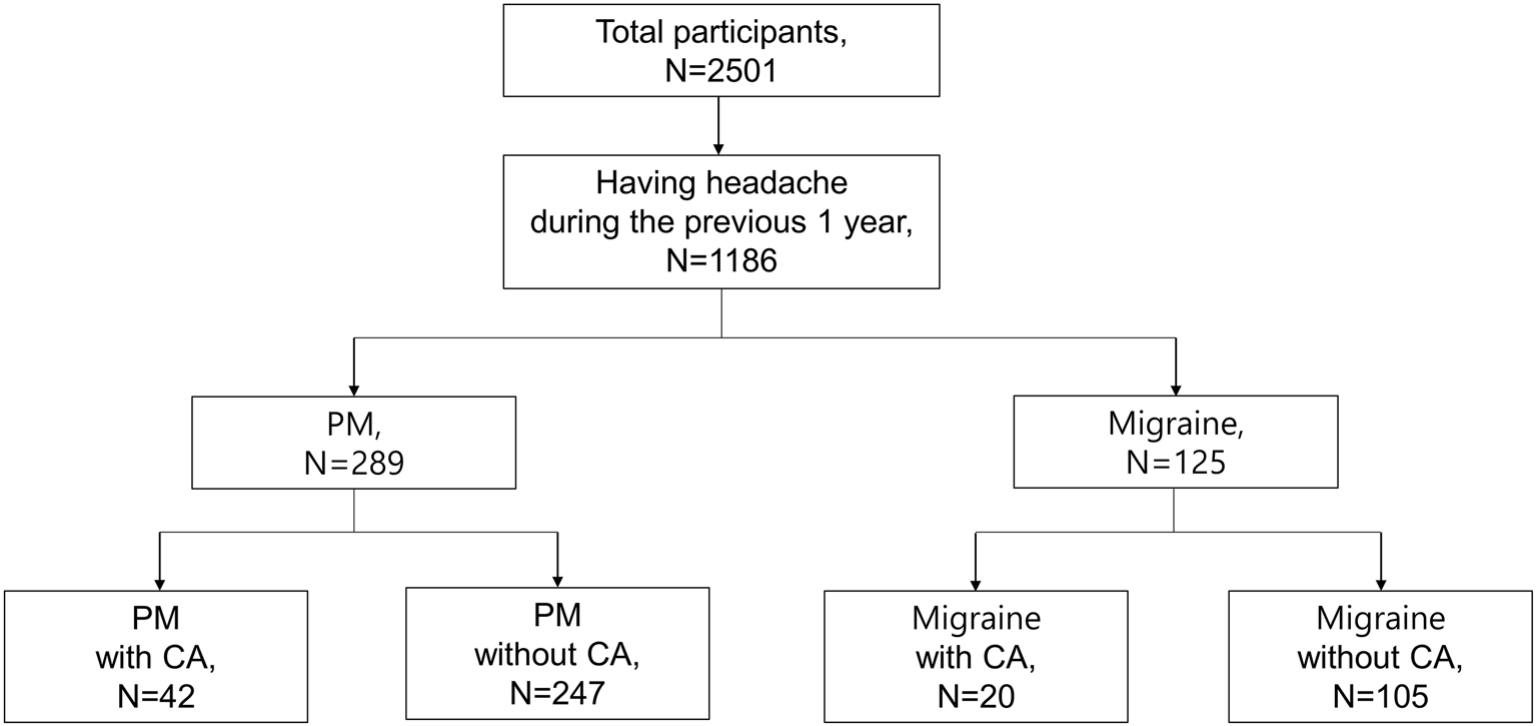
Flow chart depicting the inclusion of participants in the study. *CA* cutaneous allodynia, *PM* probable migraine.

Of the 2501 participants, 1186 responded positively to the question ‘Did you have headache during the previous one year?’. Of the 1186 respondents, 289 (11.6%) and 125 (5.0%) participants had PM and migraine, respectively (Fig. 1). Of the 289 participants with PM, 193 (66.8%) missed the typical duration (criterion B), 94 (32.5%) missed the typical headache characteristics (criterion C), and two (0.7%) missed the typical accompanying symptoms (criterion D) of migraine without aura.

There were no missing data except for PHQ-9, weight, and height. Among all participants, PHQ-9, height and weight data were missing for 3, 7, and 21 participants, respectively. Among participants with PM, the height data was missing for one participant. Among participants with migraine, weight and height data were missing for two and one participant, respectively. No PHQ-9 data was missing for any participants with migraine or PM.

### CA in individuals with PM and migraine

The responses to the ASC-12 among participants with PM and migraine are summarised in Table 2. Among participants with PM, ‘exposure to cold’ was the most common response with less than half of the time or more frequency, and ‘resting your face or head on a pillow’ was the next most frequent response. The items ‘wearing a necklace’ and ‘wearing earrings’ demonstrated the least frequencies. Of 289 participants with PM, 12 (4.1%), 15 (5.2%), and 15 (5.2%) participants were identified with mild, moderate, and severe CA, respectively. The prevalence of CA among participants with PM missing the typical duration was significantly higher than that among participants with PM missing the typical characteristics of headache (19.2% vs. 5.3%, respectively, *p* = 0.002).

**Table 2:** Responses to the Allodynia Symptom Checklist-12 by individuals with probable migraine and migraine.

Question: How often do you experience increased pain or an unpleasant sensation on your skin during your most severe type of headache when you engage each of the following?	Probable migraine, N = 289					Migraine, N = 125				
	Never, N (%)	Rarely, N (%)	Less than half, N (%)	Half the time or more, N (%)	N/A, N (%)	Never, N (%)	Rarely, N (%)	Less than half, N (%)	Half the time or more, N (%)	N/A, N (%)
Combing hair	176 (60.9)	75 (26.0)	16 (5.5)	12 (4.2)	10 (3.5)	75 (58.4)	35 (28.0)	6 (4.8)	8 (6.4)	3 (2.4)
Pulling your hair back	160 (55.4)	81 (28.0)	16 (5.5)	8 (2.8)	24 (8.3)	67 (53.6)	33 (26.4)	6 (4.8)	6 (4.8)	13 (10.4)
Shaving your face	116 (40.1)	19 (6.6)	12 (4.2)	7 (2.4)	135 (46.7)	48 (38.4)	12 (9.6)	4 (3.2)	3 (2.4)	58 (10.4)
Wearing eyeglasses	103 (35.6)	28 (9.7)	12 (4.2)	7 (7.4)	139 (48.1)	34 (27.2)	16 (12.8)	4 (3.2)	0 (0.0)	71 (56.8)
Wearing contact lens	86 (29.8)	14 (4.8)	11 (3.8)	2 (0.7)	176 (60.9)	32 (25.6)	6 (4.8)	2 (1.6)	0 (0.0)	85 (68.0)
Wearing earrings	144 (49.8)	29 (10.0)	9 (3.1)	0 (0.0)	107 (37.0)	59 (47.2)	10 (8.0)	1 (0.8)	0 (0.0)	55 (44.0)
Wearing a necklace	152 (52.6)	23 (8.0)	12 (4.2)	2 (0.7)	100 (34.6)	65 (52.0)	10 (8.0)	1 (0.8)	0 (0.0)	49 (39.2)
Wearing tight clothing	150 (51.9)	60 (20.8)	17 (5.9)	9 (3.1)	53 (18.3)	64 (51.2)	17 (13.6)	8 (6.4)	3 (2.4)	33 (26.4)
Taking a shower	184 (63.7)	68 (23.5)	21 (7.3)	9 (3.1)	7 (2.4)	79 (63.2)	35 (28.0)	7 (5.6)	0 (0.0)	4 (3.2)
Resting your face or head on a pillow	169 (58.5)	83 (28.7)	26 (9.0)	7 (2.4)	4 (1.4)	59 (47.2)	48 (38.4)	6 (4.8)	8 (6.4)	4 (3.2)
Exposure to heat	162 (56.1)	96 (33.2)	25 (8.7)	2 (0.7)	4 (1.4)	64 (51.2)	46 (36.8)	6 (4.8)	4 (3.2)	5 (4.0)
Exposure to cold	135 (46.7)	113 (39.1)	29 (10.0)	8 (2.8)	4 (1.4)	56 (44.8)	46 (36.8)	12 (9.6)	6 (4.8)	5 (4.0)

ASC-12: Allodynia Symptom Checklist-12, N/A: not applicable.

**Figure 2.**
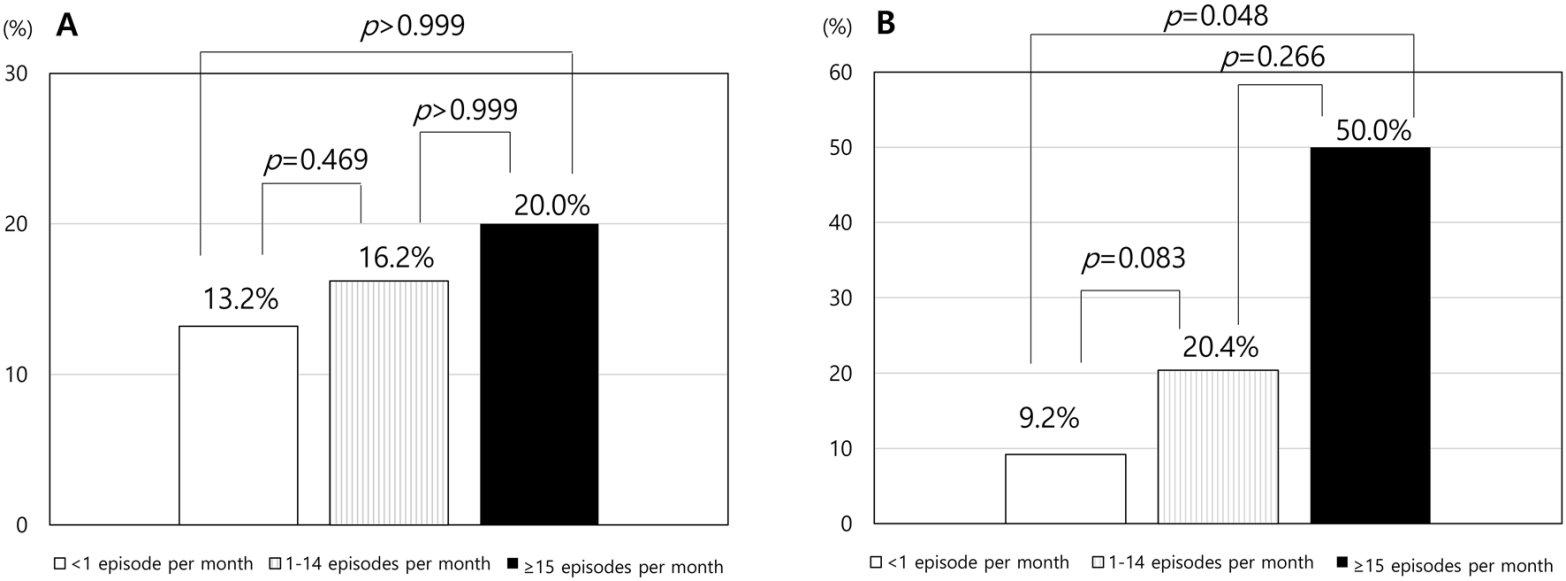
Prevalence of cutaneous allodynia in participants with probable migraine (**A**) and migraine (**B**) according to headache frequency per month.

Among in participants with migraine, CA was most frequently reported with ‘exposure to cold’ (less than half of the time or half of the time or more), followed by ‘combing hair’ and ‘resting your face or head on a pillow’. The items ‘wearing a necklace’ and ‘wearing earrings’ demonstrated the least frequent positive responses. Finally, 11 (8.8%), seven (5.6%), and two (1.6%) participants with migraine were identified with mild, moderate, and severe CA, respectively. There was no significant difference in the prevalence of CA among participants with PM according to the headache frequency (< 1 episode per month, 1–14 episodes per month, and ≥ 15 episode per month *p* = 0.725, Fig. 2A). Conversely, there was significant differences in the prevalence of CA among participants with migraine according to headache frequency (*p* = 0.017). However, post hoc analyses revealed no significant difference among the three groups (Fig. 2B). The overall prevalence of CA was not significantly different between participants with PM and those with migraine (14.5% vs. 16.0%, respectively, *p* = 0.701).

### Clinical characteristics of PM and migraine according to the presence of CA

Headache frequency per month, headache intensity (Visual Analogue Scale), impact of headache (HIT-6), and disability (MIDAS) were significantly higher among participants with PM and migraine combined with CA than among those without CA. Anxiety and depression were more prevalent among both participants with PM and migraine with CA than among those without CA. Considering participants with migraine, anxiety was more severe among participants with migraine with CA than among those without CA. Nevertheless, the severity of depression was not significantly different according to the presence of CA. Photophobia and phonophobia were more prevalent among participants with PM combined with CA; however, there was no significant difference in photophobia and phonophobia according to the presence of CA among participants with migraine (Table 3).

**Table 3:** Clinical characteristics of probable migraine and migraine according to the presence of cutaneous allodynia.

	PM			Migraine		
	PM with CA, N = 42	PM without CA, N = 247	*p*-value^†^	Migraine with CA, N = 20	Migraine without CA, N = 105	*p*-value^†^
Headache frequency per month	3.3 ± 4.3	1.8 ± 3.6	0.017	5.6 ± 8.4	2.1 ± 3.5	0.857
Headache intensity (Visual Analogue Scale)	6.0 (4.0–7.0)*	5.0 (3.0–6.0)*	0.002	7.0 (6.0–7.0)*	6.0 (5.0–7.0)*	0.033
Attack duration (hours)	2.2 ± 0.9	1.5 ± 1.1	< 0.001	9.1 ± 7.8	7.6 ± 6.6	
Unilateral pain	19 (45.2)	145 (58.6)	0.097	14 (70.0)	73 (69.5)	0.937
Aggravation by movement	27 (64.3)	86 (34.8)	< 0.001	11 (55.0)	48 (45.7)	0.501
Pulsating quality	31 (73.8)	197 (86.4)	0.337	15 (75.0)	87 (82.8)	0.547
Nausea	38 (90.5)	230 (93.1)	0.467	19 (95.0)	93 (88.6)	0.448
Vomiting	19 (45.2)	117 (47.4)	0.828	12 (60.0)	44 (41.9)	0.168
Photophobia	25 (59.5)	67 (27.1)	0.017	8 (40.0)	37 (35.2)	0.755
Phonophobia	29 (69.0)	112 (45.3)	0.007	11 (55.0)	55 (52.4)	0.917
Osmophobia	21 (50.0)	95 (38.5)	0.202	7 (35.0)	41 (39.0)	0.624
Depression (PHQ-9 score) ≥ 10	22 (52.4)	40 (16.2)	< 0.001	7 (35.0)	17 (16.2)	0.047
PHQ-9 score	11.1 ± 5.5	7.8 ± 3.5	0.150	8.6 ± 4.9	7.9 ± 3.7	0.520
Anxiety (GAD-7 score) ≥ 7	14 (33.3)	13 (5.3)	< 0.001	9 (45.0)	11 (10.5)	< 0.001
GAD-7 score	4.7 ± 5.0	2.0 ± 2.6	0.312	6.6 ± 5.4	2.2 ± 2.8	< 0.001
HIT-6 score	56.3 ± 7.2	48.3 ± 8.0	< 0.001	61.5 ± 9.2	51.4 ± 7.9	< 0.001
MIDAS score	1.0 (0.0–10.0)*	0.0 (0.0–1.0)*	0.029	5.0 (2.0–13.8)*	0.0 (0.0–2.0)*	< 0.001
Body mass index	23.1 ± 2.4	23.0 ± 2.6	0.625	22.7 ± 2.5	22.8 ± 2.5	0.116

PM: probable migraine, CA: cutaneous allodynia, MIDAS: migraine disability assessment, HIT-6: Headache Impact Test-6, PHQ-9: Patient Health Questionnaire-9, GAD-7: Generalized Anxiety Disorder-7.

*Median and interquartile range.

^†^Compared using linear regression analyses with age and sex (year, continuous) adjustment.

### Factors associated with CA in PM and migraine

For participants with PM, univariable analyses revealed that moderate and severe headache intensity, anxiety, and depression were significantly associated with CA. In multivariable analyses, moderate and severe headache intensity, anxiety, and depression were significantly associated with CA (Table 4).

**Table 4:** Factors associated with cutaneous allodynia in individuals with probable migraine in univariable and multivariable analyses.

	Probable migraine		Migraine	
	Univariable OR, 95% CI	Multivariable OR, 95% CI	Univariable OR, 95% CI	Multivariable OR, 95% CI
Sex (Female)	1.4 (0.7–2.7)	1.4 (0.6–3.2)	1.3 (0.5–3.5)	1.0 (0.3–3.8)
**Age (year)**				
20–29	REF	REF	REF	REF
30–39	1.1 (0.4–3.5)	1.5 (0.4–5.6)	1.4 (0.3–6.4)	1.1 (0.2–5.9)
40–49	0.6 (0.2–1.7)	0.8 (0.2–2.9)	1.4 (0.3–6.8)	0.5 (0.1–3.3)
50–59	0.9 (0.3–2.5)	1.2 (0.3–2.9)	0.5 (0.07–3.4)	0.2 (0.2–1.7)
60–69	0.6 (0.2–2.0)	0.4 (0.1–2.3)	1.3 (0.3–7.8)	0.2 (0.1–4.9)
> 70	1.3 (0.4–3.8)	1.4 (0.3–8.2)	0.0 (0.0–0.0)*	0.0 (0.0–0.0)*
**Educational level**				
High school	REF	REF	REF	REF
Middle school or less	1.6 (0.6–4.2)	1.6 (0.4–6.3)	0.0 (0.0–0.0)^†^	0.0 (0.0–0.0)^†^
College or more	1.3 (0.6–2.7)	0.9 (0.3–2.4)	0.9 (0.3–2.4)	0.4 (0.1–1.5)
**Headache frequency per month**				
< 1	REF	REF	REF	REF
1–14	1.3 (0.7–2.5)	0.6 (0.2–1.3)	2.5 (0.9–7.3)	1.0 (0.3–3.8)
≥ 15	1.6 (0.2–15.4)	1.1 (0.1–11.9)	9.8 (1.6–59.9)	2.6 (0.4–18.6)
**Headache intensity**				
Mild	REF	REF	REF	REF
Moderate	2.7 (1.3–5.8)	2.4 (1.1–5.4)	2.5 (1.6–4.0)	0.9 (0.2–4.6)
Severe	5.7 (1.9–16.8)	4.0 (1.1–13.9)	8.5 (4.4–16.1)	2.6 (0.4–18.6)
Anxiety (GAD-7 score ≥ 7)	9.0 (3.8–21.1)	5.2 (1.7–16.3)	11.3 (2.5–52.4)	11.6 (2.0–67.3)
Depression (PHQ-9 score ≥ 10)	5.7 (2.8–11.4)	3.3 (1.5–7.6)	2.8 (1.0–8.0)	1.1 (0.2–5.3)

OR: odds ratio, CI, confidence interval, GAD-7: Generalized Anxiety Disorder-7, PHQ-9: Patient Health Questionnaire-9. REF: Reference.

*No participant with migraine aged > 70 had cutaneous allodynia.

^†^No participant with migraine had an educational level of middle school or less.

Univariable analyses in participants with migraine indicated that ≥ 15 headaches per month, moderate and severe headache intensity, anxiety, and depression were significantly associated with CA. Multivariable analyses revealed that only anxiety was significantly associated with CA (Table 4).

## Discussion

The primary findings of the present study were the following: (1) approximately one-sixth of participants with PM and migraine experienced CA, and the prevalence of CA was not significantly different between those with PM and those with migraine; (2) participants with PM and migraine with CA experienced more severe symptoms and a greater impact of headache and disability than those without CA; and (3) headache intensity, anxiety, and depression were significant factors of CA in participants with PM. In participants with migraine, anxiety was a significant factor of CA.

Allodynia is classified as mechanical dynamic, mechanical static, and thermal allodynia. These types differ in terms of the transmission nerve fibres and nociceptors^30,31^. Each item of the ASC-12 comprised three types of CA. ‘Exposure to cold’ and ‘resting your face or head on pillow’ corresponded to thermal allodynia, and ‘combing hair’ and ‘pulling your hair back’ items corresponded to mechanical dynamic allodynia^5^. High positive response rate to items of thermal and mechanical dynamic allodynia among individuals with migraine was previously reported in a Brazilian study^32^. The present study found that ‘exposure to cold’ and ‘resting your face or head on pillow’ were the most frequently reported positive items among both individuals with PM and migraine. ‘Combing hair’ and ‘pulling your hair back’ was the next most frequent positive response. The present study is the first to identify a high for positive response rate for items corresponding to thermal and mechanical dynamic allodynia in individuals with PM, which is comparable to that in individuals with migraines.

We included participants with PM and migraine who had at least one headache attack during the preceding year. Nevertheless, some individuals with PM and migraine had a headache frequency of < 1 attack per year^33^. These individuals may not have been classified as having migraine and PM in our study. Participants with migraine and PM with a headache frequency of < 1 attack per year may have different prevalence and clinical features of CA compared to those with a frequency of ≥ 1 attack per year. Although most individuals with migraine had a headache frequency of ≥ 1 attack per year, the findings in the present study were only applicable to PM and migraine, with headache a headache frequency of ≥ 1 attack per year^33^.

Previous studies have shown that a significant proportion of individuals with migraine had CA. The American Migraine Prevalence and Prevention (AMPP) study, a large population-based study in the USA, reported that 62% of individuals with migraine had CA^7^. Migraine in America Symptoms and Treatment (MAST) study, another American large population-based study, reported that the prevalence of CA in individuals with migraine was 40%^6^. A Dutch cohort study revealed that CA was present in 70% of individuals with migraine^8^. The present study found that 16.0% of individuals with migraine had CA. These values were lower than those reported in previous studies in Western countries but results were comparable to those reported in a Korean clinic-based study, which found that 14.5% of individuals with migraine had CA^19^. One possible explanation for the lower prevalence of CA in the present study is the difference in migraine symptoms in Asian countries. Symptoms are milder in Asian countries than in Western countries. Moderate headache intensity has been reported in 30–65% of individuals with migraines in Asian countries^34,35^. In Western countries, 80–85% of individuals with migraine reported severe headache intensity^36,37^. Photophobia has been reported in 40–65% of individuals with migraine in Asian countries and 75–85% of individuals with migraine in Western countries^34,37–39^. Headache intensity and photophobia were significant predictors of CA in individuals with migraine^6^. Another possible explanation is the difference in body mass index (BMI), which is lower in Asian populations than in Western populations^40^. High BMI was reported to be a significant factor for CA in the AMPP study^5^ with obese (BMI, 30–40 kg/m^2^) and morbidly obese (BMI, ≥ 40 kg/m^2^) individuals having a higher risk of CA. In the present study, only four participants with PM and two participants with migraine were obese. Furthermore, none of the participants with migraine and PM qualified for morbid obesity. The 1-year based diagnostic strategies for PM and migraine in our study could be another possible reason for the discrepancy in the prevalence of CA between our study and previous reports. The Dutch and Brazilian studies enrolled individuals with migraine regardless of headache frequency and reported a high prevalence of CA^32,41^. The AMPP study recruited individuals who had at least one severe headache in the previous year and reported a comparable prevalence of CA^5^. The MAST study, which included individuals with migraine with monthly headache frequency, reported a slightly lower prevalence of CA than that in other studies but higher than CA prevalence found in our study^42^. The present study identified participants with PM and migraine with a headache frequency of ≥ 1 attack per year and reported a lower prevalence of CA compared to previous studies. Ethnic differences could be another possible explanation. It has been reported that pain sensitivity varies among ethnic groups^43^. Further studies in different migraine populations are required for a better understanding of the prevalence and contributing factors of CA.

In the present study, headache intensity, disability, and impact of headache were higher among participants with PM and migraine combined with CA than among those without CA. The close associations of CA with symptom severity and chronicity have been previously reported in migraine^5,7^. The present study provides evidence that such an association is also present between CA and PM.

In the present study, anxiety and depression were identified as significant factors for CA in individuals with PM. A significant association of anxiety and depression with CA has been reported previously. Kao et al. reported that anxiety was a significant factor of CA using multivariable regression analyses. Furthermore, comorbid anxiety and depression were also associated with CA severity^44^. Louter et al. reported that CA was associated with higher prevalence of depression among individuals with migraine^41^. CA, anxiety, and depression were significant risk factors of CM transformation from episodic migraine (EM)^8,45^. CM has a higher prevalence in the presence of anxiety, depression, and CA than EM^7^. Therefore, our findings corroborate existing evidence that supports the significant association of anxiety and depression with CA, suggesting shared pathophysiological mechanisms with anxiety and depression. Biogenic amines may be involved in a possible common mechanism^46^. Allodynia is a characteristic of FM, which is a chronic condition of widespread pain^47^. In an animal model of FM, decreased tactile threshold correlated with depressive behaviors^48^. The animal model demonstrated a decreased level of biogenic amines including dopamine, 5-hydroxytryptoptamine, and norepinephrine in the spinal cord, thalamus, and prefrontal cortex^48^.

The prevalence of migraine in the present study was lower than that in previous Western studies. The prevalence of migraine in Asian countries is 3–10%, which is lower than that in Western countries reported as 11–18%^28^. Therefore, migraine prevalence in the present study was similar to that in previous Asian studies. The reported prevalence of PM ranges widely (USA, 4.5%; Singapore, 6.2%; France, 10.0%; Korea. 11.5%; England: 14.6%)^14,15,17,49^; therefore, the prevalence of PM in the present study was broadly similar to that reported in previous studies.

The present study has several limitations. Firstly, we used the ASC-12 in the evaluation of CA. The gold standard for the assessment of CA is QST; however, this requires specialised equipment and is difficult to conduct in clinical practice and epidemiological studies. The ASC-12 was previously validated in comparison with QST^8^. It was originally written in English and has been translated into Portuguese, Spanish and Korean^32,50,51^. The Korean translation was constructed through forward and backward translation by a translator and reliability was assessed by calculating internal consistency of items. Portuguese and Spanish translations of the ASC-12 were not validated in comparison with QST, but studies using these two translations revealed similar prevalence of CA among individuals with migraine compared to studies using the English version ASC-12^32,50^. Using a Korean translation of the ASC-12, which was not fully validated, could be a limitation of our study^52^. Secondly, we did not evaluate cooperation rate in our survey. We conducted a headache survey in 2009 using the same sampling method, except for target sample size, and involving the same social research company, Gallup Korea^16^. Cooperation in the 2009 survey was 38.6% and we assume that the cooperation rate in this study may be comparable. The distribution of age, sex and educational level in this study was similar to that of the total population of Korea in the 2009 survey and KSHS. Furthermore, the one-year prevalence of migraine in the 2009 survey (6.1%) and the present study (5.0%) was similar to the reported prevalence in Asian countries^53^. Finally, we did not evaluate the use of medication among participants. Some types of medication for migraine prevention, such as serotonin-norepinephrine reuptake inhibitors and anticonvulsants, may relieve CA^54–56^. Further studies including medication use are required to provide accurate information of CA in patients with migraine and PM.

The present study has several strengths. Firstly, we used a two-stage clustered random sampling method proportional to the distribution of the total population of Korea. Furthermore, the estimated sampling error was low. This approach allowed us to successfully assess CA in individuals with migraine and PM in a population-based setting. Secondly, in the present study, we analysed the responses of 12 items in addition to the total ASC-12 score. We found that ‘exposure to cold’, ‘resting your face or head on a pillow’, and ‘combing hair’ were the most frequent responses given by participants with migraine and PM. Thirdly, our study used questionnaires that were specialized validated in the Korean language for assessing migraine, anxiety and depression. This process allowed us to accurately evaluate migraine, PM, anxiety and depression.

In conclusion, approximately one-sixth of individuals with PM experienced CA in a general-population-based sample in Korea. The prevalence of CA was not significantly different between participants with PM and those with migraine. Anxiety, depression, and high frequency of headaches were significant factors of CA among participants with PM.

## Data Availability

The data used in this study are available from the corresponding author on reasonable request.
